# Chronic Kidney Disease of Unknown Etiology: A Global Health Threat in Rural Agricultural Communities—Prevalence, Suspected Causes, Mechanisms, and Prevention Strategies

**DOI:** 10.3390/pathophysiology31040052

**Published:** 2024-12-09

**Authors:** Zineb Ben Khadda, Haitam Lahmamsi, Yahya El Karmoudi, Said Ezrari, Laila El Hanafi, Tarik Sqalli Houssaini

**Affiliations:** 1 Laboratory of Epidemiology and Research in Health Sciences, Faculty of Medicine and Pharmacy, Sidi Mohammed Ben Abdellah University, PO 1893, Km 2200, Route Sidi Harazem, Fez 30000, Morocco; tarik.sqalli@usmba.ac.ma; 2 Laboratory of Microbial Biotechnology and Bioactive Molecules, Faculty of Science and Technology, Sidi Mohamed Ben Abdellah University, Route Immouzer BP 2202, Fez 30000, Morocco; haitam.lahmamsi@usmba.ac.ma; 3 Laboratory of Ecology, Systematics, Conservation of Biodiversity, LESCB URL-CNRST N° 18, Faculty of Sciences, Abdelmalek Essaadi University, PO 2121 M’Hannech II, Tetouan 93002, Morocco; yahyaelkarmoudi@gmail.com; 4 Microbiology Unit, Laboratory of Bioresources, Biotechnology, Ethnopharmacology and Health, Faculty of Medicine and Pharmacy Oujda, Mohammed First University, PO 4867 Oujda University, Oujda 60049, Morocco; 5 Department of Biology, Laboratory of Functional Ecology and Engineering Environment, Sidi Mohamed Ben Abdellah University, Route Immouzer BP 2202, Fez 30000, Morocco; laila-elhanafi@hotmail.fr; 6 Department of Nephrology, Hassan II University Hospital, BP 1835, Atlas, Road of Sidi Harazem, Fez 30000, Morocco

**Keywords:** chronic interstitial nephritis in agricultural communities, kidney disease of unknown etiology, CKDu, CINAC, pesticides, heat stress

## Abstract

Chronic Kidney Disease of Unknown Etiology (CKDu) is a worldwide hidden health threat that is associated with progressive loss of kidney functions without showing any initial symptoms until reaching end-stage renal failure, eventually leading to death. It is a growing health problem in Asia, Central America, Africa, and the Middle East, with identified hotspots. CKDu disease mainly affects young men in rural farming communities, while its etiology is not related to hypertension, kidney stones, diabetes, or other known causes. The main suspected causal factors are heat-stress, dehydration, exposure to agrochemicals, heavy metals and use of hard water, infections, mycotoxins, nephrotoxic agents, altitude, and genetic factors. This review gives an overview of CKDu and sheds light on its medical history, geographic distribution, and worldwide prevalence. It also summarizes the suspected causal factors, their proposed mechanisms of action, as well as the main methods used in the CKDu prior detection and surveillance. In addition, mitigation measures to reduce the burden of CKDu are also discussed. Further investigation utilizing more robust study designs would provide a better understanding of the risk factors linked to CKDu and their comparison between affected regions.

## 1. Introduction

Chronic kidney disease (CKD) poses a significant worldwide public health challenge, being considered an independent risk factor for cardiovascular events and may be a key determinant of adverse health outcomes. In the early stages, CKD does not show any symptoms until it reaches advanced levels; unfortunately, this epidemic is widespread worldwide, affecting between 8% to 16% of the world’s population [1].

Indeed, observational studies found that the prevalence of CKD is higher in low- and middle-income developing nations compared with high-income industrialized countries [2].

Globally, CKD is most commonly attributed to diabetes and/or hypertension [1].; however, there appears to be an alarming rise in the incidence and prevalence of CKDu, with no known risk factors [3].

**Figure 1:**
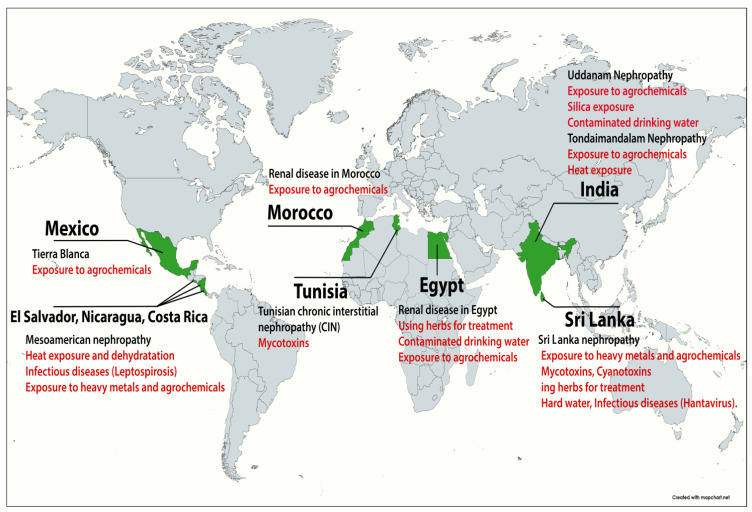
Geographical distribution of CKDu. in the Mesoamerican region [11,12], in Srilanka [13], in Morocco [14], in Tunisia [15], in Egypt [16], in India [17], and in Mexico [7].

CKDu has been recorded in tropical and subtropical climates, particularly in several Central American nations, the dry zone of Sri Lanka, certain states of India, Japan, and some North-African countries such as Tunisia and Egypt [4,5,6,7] (Figure 1). Several names were attributed to this disease depending on the region where it was diagnosed, such as Mesoamerican nephropathy [8], Uddanam nephropathy [9], Tondaimandalam nephropathy [10], etc.

Since its emergence, scientists have been trying to understand the disease’s behavior and its risk and causative factors to provide a promising solution. Epidemiological studies indicate that heat stress, exposure to environmental contaminants, and agriculture-associated dehydration [18,19]. The contribution of synergistic effect between environmental factors alongside a genetic predisposition was also suggested by several studies either in the initiation or the progression of renal dysfunction [20].

Due to all this, the precise causes of this disease are a subject of considerable debate, leaving the origin of the disease a mystery that needs to be unveiled. Kidney function silently deteriorates to end-stage kidney disease (ESKD), which is often fatal in areas where only a few patients can afford kidney replacement therapy, representing a serious public concern [21].

This review results from a general analysis of the CKDu situation, covering medical definitions, its worldwide prevalence, and suspected risk factors; however, knowledge of the different possible pathways of various suspected risk factors is essential. A full evaluation of the molecular pathways involved remains an important gap in our understanding. To this end, we examine the potential mechanisms that could mediate the renal effects of exposure to heat stress and environmental toxins. Understanding these mechanisms can guide us to take preventive actions. We also report the most promising biomarkers for early detection of chronic kidney disease in order to develop new treatment strategies.

### CKDu: Definition, Clinical Profile and Histopathology

CKDu, also called Chronic Interstitial Nephritis in Agricultural Communities (CINAC), and previously described as Mesoamerican Nephropathy, is a disease primarily affecting young men belonging to agricultural communities in specific areas with high prevalence, and occasionally women and adolescents; its etiology is not related to diabetes, hypertension, glomerulonephritis, pyelonephritis, kidney stones, snakebite or other known causes [22].Most CKD patients are asymptomatic in the early stages. Symptoms start appearing as early as stage 2 when the frequency and intensity of symptoms progress as the disease progresses [23]. The commonly reported symptoms are arthralgia, asthenia, muscle cramps, decreased libido, and fainting [24]. Urinalysis shows no or minimal proteinuria (<1 g per 24 h), low red blood cell and leukocyte counts, and sometimes amorphous urate crystals. Serum electrolyte abnormalities may involve hypokalemia, hyponatremia, and hypomagnesemia linked with increased urinary electrolyte losses [25]. Hyperuricemia is common but not essential for diagnosis [13].

Renal ultrasound showed echogenicity, decreased cortico-medullary ratio, and border irregularity in late CKD. In contrast, normal blood flow in either the parenchyma or renal vessels was shown in renal Doppler, with no malignant lesions when the urinary tract and bladder ultrasound was performed. The ECG and blood pressure are normal or slightly elevated in almost all patients [26].

In agricultural communities around the world, histological studies of chronic interstitial nephritis have revealed proximal tubular atrophy, tubulointerstitial fibrosis, an inflammatory response, and thickening and wrinkling of tubular basement membranes [27,28,29]. In proximal tubular epithelial cells, enlarged dysmorphic lysosomes (>1.2 μm in largest diameter) with electron-dense aggregates (or clusters of smaller lysosomes) were found very often when subjected to electron microscopy [30].

## 2. Geographical Distribution and Prevalence of CKDu

### 2.1. Mesoamerican Nephropathy (MeN)

Cases of chronic renal failure of unknown etiology, referred to as MeN, have been epidemic in the Mesoamerican region since 2002. This region encompasses El Salvador, western Nicaragua, northwestern Costa Rica, Southeastern Mexico, and Guatemala [31]. According to recent studies, MeN is now the most significant contributor to premature death among young adults in areas where it is endemic [32]. Population-based studies have recorded a prevalence of MeN ranging from 17.9% to 21.1% in Central America [23] This disease is commonly found in rural farming communities located in low-lying coastal regions characterized by high humidity and extremely high ambient temperatures, where other economic activities, such as fishing and mining, are also present [33]. In Central America, the prevalence of CKD varies significantly between regions and genders. Almaguer et al. [23] found that in low-lying farming communities, mainly in sugarcane-growing areas, the prevalence of CKD among men ranged from 16.9–20.1%, while among women, it was 4–8.1%. At higher altitudes, the prevalence was lower, with 0–7.5% in males and 1.25–4% in females.

Regions characterized by high humidity and extremely high temperatures, where economic activities such as fishing and mining also thrive.

In El Salvador, the regional prevalence ranged from 7.1% in the para-central region, 5.2% in the east region, and 2% in the center region [34]. In Nicaragua, the regional prevalence varied between 8% and 10% [11] found that the prevalence was higher among banana and sugar cane plantation workers. In Costa Rica, in the Guanacaste province, the number of new cases of CKDu all involve young men working as sugar cane cutters [35]. Throughout the period ranging from 1970 to 2012, CKD mortality rates in men increased from 4.4 to 38.5 per 100,000, which is much more compared to 3.6 to 8.4 in the rest of Costa Rica, while in women, it ranged from 2.3 to 10.7 per 100,000 against 2.6 to 5.0, respectively [36].

### 2.2. Sri Lankan Nephropathy

The north-central province of Sri Lanka is considered an endemic region of CKDu, where up to 10% of the adult population is affected [25]. More than 70% of cases are rice farmers, in particular paddy cultivation, while the rest depend on market gardening [23]. The higher CKDu or CINAC prevalence in Sri Lanka was recorded in Padaviya, Sripura, Dehiattakandiya, Girandurukotte, Medawachchiya, Nikawewa, Kabithigollawa, Giribawa, Polpithigama, Wilgamuwa, Mahiyanganaya, Rideemaliyadda, Welioya, Mahawa, and Tantirimale [12,37].

### 2.3. Indian CKDu

#### 2.3.1. Uddanam Nephropathy

The name Uddanam is derived from the word “Udhyanam”, meaning garden, which is characteristic of the Andhra Prades Indian state; it is a coastal region bordered by mountains (the Eastern Ghats), and rich in vegetation, including coconuts, cashews, jackfruits and rice fields. CKDu has become the second most common type of CKD after diabetic nephropathy in India [38]. A cross-sectional study reported an 18.23% prevalence of CKD in a rural population in Uddanam region [39].. A high prevalence of CKDu (16%) in the Uddanam region, India’s rural communities, was reported. Hence, patients with CKD diagnosed had no cause related to the already known factors such as hypertension or diabetes; this study considered CKDu as a real threat to public health in Uddanam [40]. A comprehensive cross-sectional screening in the rural areas in Andhra Pradesh indicated a relatively high incidence of CKD (32.2%), particularly among male farm workers [17].

#### 2.3.2. Tondaimandalam Nephropathy

A recent study carried out in southeastern India, namely the states of Puducherry and Tamil Nadu, an area historically known as “Tondaimandalam”, revealed a high incidence of CKDu. A total of 2424 patients were analyzed, with CKD as the most common etiologic category (51.7%). Among all studies published to date on CKD, this event represents the highest percentage of CKDu reported in India. Another community screening program of 447 people showed a prevalence of CKDu of 19%, with most patients coming from a poor background and engaging in agricultural work or working in a hot climate [41,42]. As a result, the name “Tondaimandalam Nephropathy” has been proposed for this entity

### 2.4. Other CKDu Hotspots

Current information presented in many studies indicates occurrences in several other countries. Aguilar and Madero [6] have reported that Mexico may have newly identified CKDu hotspots, with Tierra Blanca being one of them. This predominantly rural region located in the eastern part of Mexico has an agricultural-based economy. The prevalence of kidney disease among Tierra Blanca residents is 25%, with 44% of those cases having no known risk factors and thus being considered CKDu cases.

In 2003, Hassen et al. [43] described chronic interstitial nephropathy (CIN) in the Tunisian population with characteristics similar to other forms of nephropathy. Khlifa et al. [15] later identified ochratoxin A, which is a type of mycotoxin that occurred naturally with nephrotoxic properties, in large amounts in human serum collected from patients with CIN of unknown etiology. Across Egypt, the prevalence of this unknown-cause disease was estimated to be 15.2%. In the governorate of El Beheira, this figure was 21.6% [44]. El Minia had a prevalence of 27%, Cairo 18.1% [45], and Manorial 32.9% [46]. Unsanitary water consumption, exposure to pesticides, and the use of herbs for treatment were identified as risk factors for CKDu [47]. During a cross-sectional study, a CKD prevalence of 6.5% was recorded in rural communities of the Fez-Meknes region, Morocco; 38.4% among them were farmers exposed to pesticides and who had no causes related to diabetes, hypertension, or other known factors [16].

## 3. Possible Etiological Factors of CKDu

Over the past two decades, a widespread outbreak of CKDu has already been reported. Numerous studies have proposed theories and conducted research to try to identify the potential causes of the disease. The main risk factors identified during these studies are agrochemicals, heavy metals, heat stress, mycotoxins, etc; however, the exact cause of CKDu remains unknown and debatable.

### 3.1. Agrochemicals

The use of agrochemicals has been widely considered a potential risk factor for the outbreak of CKDu. Valcke et al. [20] reported experimental and clinical evidence that several pesticides commonly used in various regions across the world have been linked to the development of CKDu. These pesticides, which have been identified as human nephrotoxins, include glyphosate [14], paraquat [48], cypermethrin, 2,4-Dichlorophenoxyacetic acid (2,4 D), and carbamate [49]. As such, agriculture is one of the main activities in countries affected by CKDu. A recent cross-sectional study noted a decrease in kidney function among farmers; in addition, the poisoning with organophosphate (OP) and carbamate (CA) was significantly associated with a 4.703 times higher risk of CKDu [50]. A meta-analysis conducted by Chapman et al. [51] suggested that exposure to agrochemicals and agricultural work increases the risk of CKDu.

The herbicides Glyphosate, 2,4-d, and Paraquat have been widely used and have been shown to increase the urea and uric acid levels in the blood, leading to oxidative stress in the body cells. This oxidative stress has been linked to kidney tubule cell damage, which can ultimately result in kidney failure [52]. A recent computational approach using docking and molecular dynamics simulation studies revealed that pesticides may be a possible high-risk factor for CKDu, and their metabolites have more potential to have greater associations with kidney enzymes compared with the parent pesticides [53].

### 3.2. Heavy Metals

Heavy metals are widely used in agriculture and industrial applications, including the production of pesticides, batteries, alloys, and textile dyes. Divalent metals, when present in high concentrations, can cause damage to the kidney, which is the first organ in the human body to reabsorb and accumulate them [54]; therefore, chronic exposure to these elements, including arsenic (As), cadmium (Cd), chromium (Cr), and lead (Pb), has been suspected in the CKDu onset and documented in several studies. In early attempts to identify its etiology, As was considered a likely potential risk factor for kidney disease.

The findings from a cross-sectional study of patients in Taiwan revealed a positive correlation between urinary As levels and the incidence of CKD. The study concluded that elevated levels of As could potentially increase the incidence of CKD by four times [55].

In addition, 68% of patients with CKDu have had a urinary arsenic level above the nephropathy threshold limit, suggesting a potential link between the disease and chronic arsenic toxicity. This could be of agricultural origin; no research has detected traces of arsenic in the sediments of Sri Lanka [56].

The contamination of rice, which is the main diet of the inhabitants of a hot spot of CKDu, by nephrotoxic metals, particularly Pb and Cd, has been detected in Sri Lanka [57]. In the United States, biological samples (such as serum, urine, hair, and nails) from patients with CKDu have shown higher levels of lead and cadmium compared with the reference values and to levels found in non-patients [58,59]. Assessment of toxic metal contamination situation in surface sediments of reservoirs in distinct endemic of CKDu in Sri Lanka also showed that almost all reservoirs were moderately polluted by Cd, As, Cr, and Pb, revealing a risk posed to aquatic life as well as humans via contamination of the food chain [60].

### 3.3. Dehydration/Heat Stress

Conversely, numerous studies have validated the heat stress/dehydration hypothesis, establishing it as the primary driver or essential cause of [61,62,63]. Working in excessive heat has been associated with dehydration, which leads to acute pre-renal injury (AKI). It has also been shown that working more than 6 h in the field under the sun and consuming less than 3L of water per day are factors that lead to the development of CKDu [64].

In Nicaragua, dehydration was considered a predisposing factor for chronic renal failure in agricultural workers exposed to very high temperatures, leading to volume depletion, which increases the kidneys’ sensitivity to various nephrotoxic agents [65]. MeN is often present in male farmers working in hot climates [66]. Suggesting that lower renal function in such situations correlates with dehydration and subsequent AKI, leading to CKD [6,36]. Other research has also investigated climate change as a possible risk factor related to the incidence of MeN [66,67]. Heat stress may contribute to the perpetuation of CINAC, but it is unlikely to be the sole driver because, in warmer regions (such as Cuba and northern Sri Lanka) where agrochemicals are rarely or never used, the CINAC is absent.

### 3.4. Hard Water

According to the World Health Organization (WHO), hard water is defined as water containing magnesium, iron, strontium, bicarbonate, sulfate, carbonate, calcium, or chloride anions. Several studies reported a correlation between CKDu occurrence and high groundwater hardness [68,69,70].

A study of groundwater quality in CKDu-affected areas in Sri Lanka revealed high levels of drinking water hardness, elevated cadmium concentrations, and fluoride concentrations that exceeded the WHO-recommended levels [71]. Another study showed significant relationships between hardness due to Mg and dissolved ions in water and the incidence of CKDu [72].

Substantially high ionic content was also found in drinking water, leading to higher salinity and total hardness levels than Sri Lankan standards. This indicates a significant correlation between the occurrence of CKDu and quality parameters such as the presence of phosphate ions and total dissolved solids content [73].

A common feature of groundwater in all CKDu endemic areas was high fluoride content, which can cause kidney tubular damage [74]. A relatively recent survey hypothesized that high levels of fluoride in drinking water sources in parts of Sri Lanka might be associated with the increasing prevalence of CINAC. The average fluoride content of the analyzed water samples was found to be higher than the maximum allowable levels set by the standards [75]. In addition, it has been shown that the mutual presence of fluoride, as well as the hardness of magnesium and/or cadmium in solution, could have more nephrotoxic effects than fluoride ions alone [76].

Several studies underline the harmful effects of excess fluoride on the human kidneys. Exposure to this latter disrupts collagen synthesis, induces collagen breakdown in the kidneys, and increases levels of hydroxyproline/hydroxylysine in blood and urine, which are not found in other proteins, leading to kidney dysfunction and chronic kidney disease failure [77].

Silica has been considered as another possible risk factor for CKDu. A case-control study conducted in the United States showed a positive association between silica exposure and CKD in a dose–response manner [78]. Another research study from South India showed similar results on the possible nephrotoxic impact of silica [79]. Research on animals indicates that silica exposure in mice causes both an inflammatory and fibrotic response in the kidneys [80].

### 3.5. Nephrotoxic Agents

Exposure to nephrotoxins can cause both AKI and CKD. These agents include nonsteroidal anti-inflammatory drugs (NSAIDs), antibiotics, iodinated contrast media, and chemotherapeutic drugs [81].

NSAIDs are the most improperly used nephrotoxic medicines; their risk resides in direct interference with kidney function through prostaglandin inhibition, which leads to moderate and temporary chronic kidney disease [82]. Other research has demonstrated that regardless of the medication class, whether selective or not, higher doses and longer half-lives considerably increase the potential of developing CKD [83].

Traditional and alternative remedies are widely employed for preventive and therapeutic purposes [84]. Numerous epidemiological studies have supported the harmful effects of the irrational use of medicinal plants among patients with chronic kidney disease, particularly those that may contain aristolochic acid [85,86]. The most reported adverse effect was nephropathy, especially interstitial nephritis, inducing ESKD and urothelial malignancy [87]. Renal tubular epithelial cells absorb aristolochic acid (AA) through the organic anion transporter OAT_1/3_ [88]_._ AA induces a fast increase in the intracellular Ca^2+^ concentration of renal tubular cells in response to the release of Ca^2+^ stores from the endoplasmic reticulum and the extracellular Ca^2+^ influx, which induces endoplasmic reticulum and mitochondria stress [89].

Furthermore, experiments on cultivated tubular epithelial cells have indicated that AA therapy causes increased levels of reactive oxygen species (ROS). Stress leads to caspase activation and, ultimately, apoptosis [90].

The acute phase of aristolochic acid (AA) administration is characterized by an elevation of plasma creatinine and necrosis of proximal tubular epithelial cells. During this phase, cells generate inflammatory cytokines, which contribute to the accumulation of inflammatory cells in the renal interstitium, thus creating an inflammatory infiltration composed of diverse immune cells [91]. Prolonged inflammation eventually leads to chronic interstitial inflammation, tubular atrophy, and prominent fibrosis [92].

### 3.6. Infections

Leptospirosis and hantavirus infections are important rodent-borne zoonotic diseases known to cause AKI and may progress to CKD [93,94]. Both infections share similar clinical presentations and epidemiological features, making them prone to clinical misdiagnosis and difficult to distinguish because of their non-specific symptoms [95].

Leptospira infection can induce renal fibrosis, as shown by in vitro and in vivo tests on animal models [96,97] A systematic review of observational studies conducted in Taiwan and Nicaragua revealed that the average estimated glomerular filtration rate (eGFR) was significantly lower (*p* < 0.001) in the general population with positive anti-leptospira antibodies compared with the negative controls. Additionally, among sugar cane workers with high creatinine, those who were HIV-positive had an eGFR below 60 [98].

A study conducted in an endemic area for CKDu in Sri Lanka revealed a high seroprevalence of hantavirus in patients with kidney disease, indicating a possible involvement of hantavirus in the development of kidney failure Similarly, in another cross-sectional study, levels of IgG antibodies against hantavirus were evaluated in 132 CKDu patients; among the studied group [99], 54.5% of the subjects were seropositive [100]. Other serological results indicated a notably higher prevalence of hantavirus exposure among patients with CKDu in Girandurukotte, supporting the hypothesis that hantavirus may potentially contribute to the risk of CKDu in Sri Lanka [101].

### 3.7. Altitude

At high altitudes, the prevalence of CKDu reported in several studies was much lower [36,102]. Sugarcane workers who lived and worked in coastal regions had higher SCr levels and lower eGFR than those who resided within 500 m of altitude [103]. A meta-analysis also found positive associations between lowland elevation and CKDu [104].

However, some studies have shown that living in high altitudes could be considered as kidney damage risk factor, as it can cause high altitude renal syndrome (RAS), which leads to chronic hypoxia that is characterized by hyperuricemia, polycythemia, proteinuria, and hypertension [105,106].

In Central America, Dharma-wardana [76] highlighted the potential relationship between the risk of chronic kidney failure and the high-altitude residence; for each 200 m above sea level of the residence, the subjects’ probability of chronic renal failure increased by 26% (OR 1.26 95% CI 1.15–1.38, *p* < 0.001) [76]. Another recent study conducted in Indonesia also showed that farmers living in high-altitude areas were twice as likely to have CKDu as farmers in low-altitude areas [107].

### 3.8. Mycotoxins

Mycotoxins are secondary metabolites produced by fungi that grow in rice, corn, peanuts, wheat, and other improperly stored products, which have been linked to kidney disease and other adverse health effects, including cancer [108].

In Sri Lanka, a study detecting levels of mycotoxins in urine samples from patients with CKDu revealed that in the 31 patients studied, 61.29, 93.5, and 19.4% had aflatoxins, ochratoxins, and fumonisins, respectively [109]. A comparison of ochratoxin A (OTA) levels in blood samples from dogs with CKD and healthy dogs showed that the highest incidence of OTA positivity was recorded in the CKD group (96% versus 56%) [110]; however, studies evaluating the direct relationship between mycotoxins and CKDu are limited, and further analysis is needed.

### 3.9. Genetic Factors

Genetic factors may also be important and could explain family clustering of chronic kidney disease in communities [25]. Genetic differences in ethnicities are recognized to significantly influence the prevalence and the risk of progression of CKD. For example, black Americans have a three to five times higher risk of developing kidney disease compared with white Americans [111]. Additionally, African Americans who carry the APOL1 genotype have higher rates and faster progression of CKD [112].

Whole-exome sequencing reveals genetic variants associated with CKD characterized by tubulointerstitial lesions in the north-central region of Sri Lanka [113]. In a cohort of adults, an updated genetic risk score using 53 genetic loci associated with diminished kidney function predicted a stage 3 CKD incident. When sex, age, and other risk factors were considered, every increase of 10 alleles was linked to a 37% increased risk of CKD [114].

## 4. Possible Molecular Mechanisms That Could Induce Renal Injury

**Figure 2:**
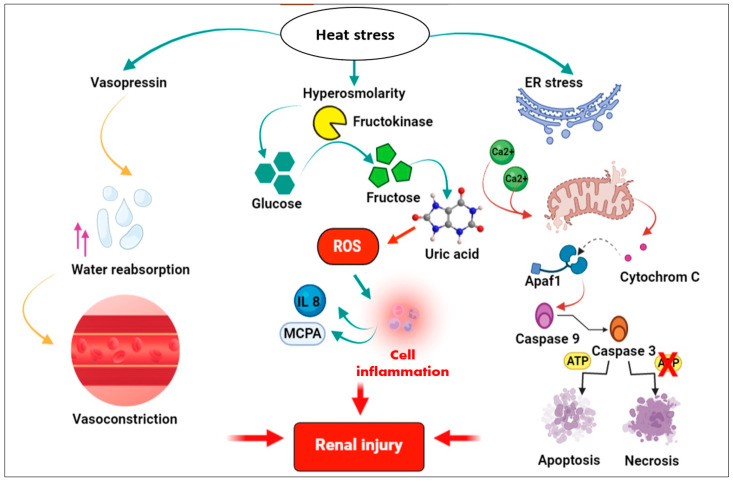
Heat stress-induced renal injury. Heat stress-mediated hyperosmolarity activates vasopressin; released vasopressin increases vasoconstriction. The activation of the polyol pathway converts glucose to fructose and increases ROS levels, activating the inflammatory pathway. ER and mitochondrial stress also activate the cell death pathway. This figure was exported under a paid subscription with BioRender.

**Figure 3:**
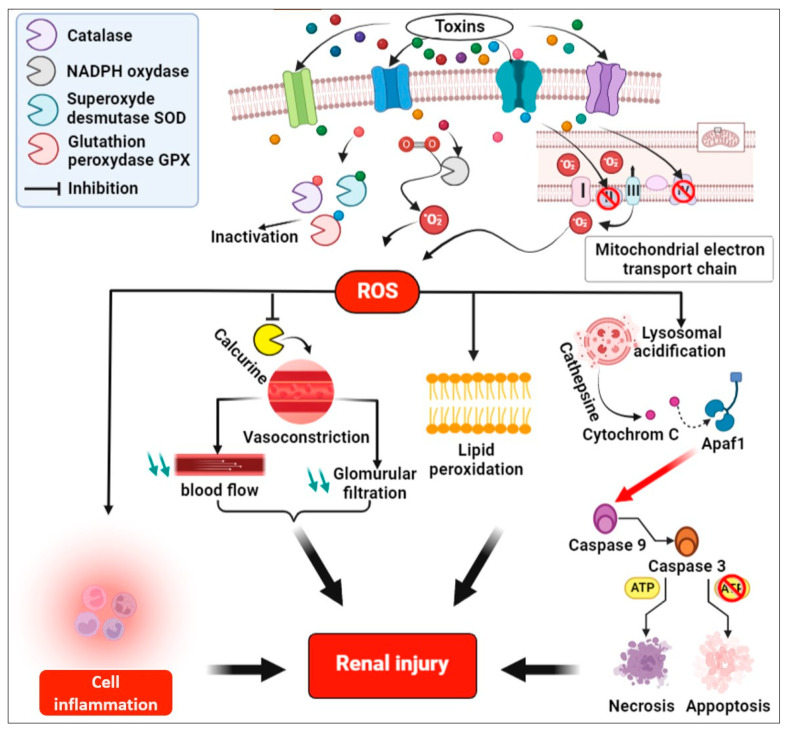
Common pathways of toxins induced renal injury. Alteration in the mitochondrial electron transport chain and main oxidases such as NADPH oxidase led to excessive ROS production with toxins exposure. Increased ROS generation activates and induces some pathways, such as the caspase pathway, inhibition of lysosome enzymatic function, lipid peroxidation, cell death, and inflammation. This figure was exported under a paid subscription with BioRender.

This disease’s etiology is highly debated, but it is generally accepted that CKDu is a disease that can be induced by environmental factors due to a combination of toxicities: heavy metals, fluoride, hard water, heat stress, and pesticides. Supporting the hypothesis that the etiology of this disease could be multifactorial. Two main hypotheses dominate in the current etiological debate: where the first suggests the association of CKDu with heat stress and periodic dehydration (Figure 2), and the second suggests the association of CKDu with repeated, long-term exposure to environmental toxins (Figure 3).

### 4.1. Molecular Mechanisms of Heat Stress and Dehydration-Induced Renal Injury

It has been proposed that working in excessive heat has been associated with hyperosmolarity, which releases vasopressin into the blood by stimulation of the posterior pituitary, which increases water reabsorption in the kidney and vasoconstriction, causing kidney tissue damage [61,115].

Hyper-osmolarity also activates aldose reductase in the proximal tubule, converting glucose into fructose by fructokinase [67]. Moreover, the activation of the polyol pathway leads to the generation of ROS, stimulating the inflammatory pathway, which generates inflammatory chemokines such as MCP-1. This latter is implicated in local tubular damage and inflammation [116], which may be further promoted by the tubular metabolism of fructose and lead to uric acid production [117]. Which, in turn, may also be associated with renal damage, including glomerular damage and tubulointerstitial fibrosis [61].

Heat stress can then induce oxidative stress by reducing mitochondrial metabolic oxidative capacity, leading to a clear alteration in the pattern of antioxidant enzymatic activities and the depletion of antioxidant reserves [118].

Several studies have shown that hyperthermia induces apoptotic cell death through a mitochondria-mediated pathway. In particular, bovine mammalian epithelial cells [119], U937 human myelomonocytic lymphoma cells [120], and calf kidney cells [121]. The permeability transition pore (PTP) mainly consists of the voltage-gated anion channel in the outer mitochondrial membrane [122]. The activation of this ion channel complex triggers permeabilization of the mitochondrial membrane, which leads to depolarization of the membrane potential and may also result in the flux of ions and water molecules into the mitochondria [123].

The endoplasmic reticulum (ER) provides areas of close contact with the mitochondria. Thus, within the ER membrane, the inositol triphosphate receptor, IP3R, participates in the passive release of Ca^2+^, overloading the mitochondria with Ca^2+^ and altering their functions, in particular oxidative phosphorylation. This alteration of oxidative phosphorylation leads to ROS production [124].

Mitochondrial Ca^2+^ and ROS overload induced by hyperthermia result in matrix swelling and outer mitochondrial membrane rupture (OMM). The resulting OMM permeabilization releases apoptotic factors such as cytochrome c that bind to apoptotic protease, activating factor 1 (Apaf-1) and forming the Apaf-1/procaspase-9 apoptosome; this complex activates in turn caspase-9, then caspase-3 [125].

### 4.2. Molecular Mechanisms of Toxins Induced Renal Injury

The absorption of toxins via cellular transporters induces the production of high amounts of ROS in renal tissues via enzymatic complexes of the mitochondrial transport chain or via enzymatic complexes of electrons [54].

Electrons flow through complexes 1, 2, 3, and 4, converting 1 to 2% of the available O^2^ in the mitochondria into O^2-^, while with exposure to metals, the leakage of electrons at the level of complexes 1 and 3 increases. This leakage is due to the inhibition of cytochrome c oxidase COX activity, and these electrons then react with O^2^ and produce O^2^ radicals [126]. One of the main enzymatic complexes activated by metals is NADPH, which catalyzes the process of converting O^2^ molecules into radicals by donating electrons [127]. In addition, these metals inhibit the main antioxidant enzymes such as superoxide dismutase (SOD), glutathione peroxidase (Gpx), and catalase (CAT), leading to an additional accumulation of ROS in cells [128].

Proximal tubular cells are significant in generating ROS due to the intense oxygen consumption during ATP production, particularly through active transport mechanisms. In vivo studies on rats indicate that proximal tubules do not possess the capability to synthesize glutathione [28]. Instead, it relies on circulating glutathione to protect it from mitochondria-generated ROS. The proximal tubules are then the key regions affected by the pathology of CINAC.

Oxidative stress and free radical damage inhibit lysosome enzymatic function [129]. Lysosomes contain several enzymes that degrade cellular macromolecules during autophagic and endocytic activities. Lysosomal membrane permeabilization (LMP) can be caused by osmotic lysis or detergent action of chemicals that accumulate in the lumen of lysosomes [130]. The initiation of the lysosomal cell death pathway can occur through the liberation of cathepsins and other hydrolases from the lysosomal lumen into the cytosol [28,131].

Cathepsins participate in the proteolytic activation of pro-apoptotic proteins such as Bax, as well as in the degradation or inactivation of anti-apoptotic Bcl_2_. This process leads to the permeabilization of the mitochondrial membrane, the subsequent release of cytochrome c (Cyto c), and ultimately, cell death [129].

Some pesticides and heavy metals are chemical examples that alter lysosomal functions. Heavy metal poisoning has been found to increase mitochondrial oxidative stress and worsen damage to proteins, nucleic acids, and lipids [132].

Indeed, two metals, Cd and As, are notable examples of metals that induce and modify autophagy, which, in the case of their presence in the environment, have been associated with cellular transformation mechanisms [133]. Moreover, in the proximal tubular cells of rats treated with cadmium, an increase in the expression of LC3-II and p62 proteins was observed [134]. LC3-II is an essential protein for the elongation and closure of the phagophore, located in the membranes of auto-phagosomes, while p62 is a receptor protein present inside the auto-phagosome [135,136]. This suggests inhibition of autophagy flux, which was associated with reduced autophagosome–lysosome fusion. This is consistent with this study, which found that prolonged exposure to cadmium increased apoptosis in chicken kidneys through autophagy [137].

In the same context, the accumulation of these two proteins was also observed in female mice exposed to arsenic [138]. The nephrotoxicity of organophosphate pesticides has garnered increased attention. In laboratory animal experiments, OP nephrotoxicity has been found for diazinon [139], Malathion [140,141], Methamidophos [142], dichlorophos [143], and Chlorpyrifos [144].

Many authors consider oxidative stress to be the primary mechanism of toxicity in both in vitro and in vivo animal studies, as well as in clinical studies, regarding subchronic or chronic exposure to organophosphates (OPs) [145]. The accumulation of autophagosomes has been observed as a consequence of malathion-induced destabilization of lysosomal membranes, leading to the impairment of autophagosome–lysosome fusion [146].

Difenoconazole induces oxidative stress, inflammation, and apoptosis in carp kidneys by promoting the accumulation of ROS. This compound also enhances the transcription of apoptosis-related genes such as p53, caspase9, caspase3, and bax, while inhibiting the expression of Bcl-2. Moreover, TEM imaging revealed the formation of clearly visible autophagic lysosomes and autophagosomes [147]. The results from another study suggest that fluoride decreases lysosomal degradative capacity and triggers autophagic outflow blockade and apoptosis [148].

Furthermore, a recent study suggests that the processes of mitochondrial, lysosomal, and protein reabsorption dysregulation may be involved in chronic kidney disease of unknown etiology and could help better understand the underlying mechanisms of the disease to develop new treatment strategies [149].

The increased level of ROS in kidney tissue also leads to lipid peroxidation [150,151]. Different inflammatory pathways are also triggered by ROS, which then induces genes responsible for inflammatory molecules, in particular IL [152,153].

In addition to the epidemiological links between the use of toxins, including pesticides, and the development of CINAC, there are several mechanistic reasons that justify the need to investigate a possible etiological role through the calcineurin pathway. The calcineurin pathway is a cascade of cellular signaling involved in the regulation of various biological processes, primarily known for its role in the immune system, where it regulates the production of cytokines and the differentiation of T lymphocytes. It is mainly mediated by a protein called calcineurin, a calcium-dependent phosphatase [154,155].

Normally, when T lymphocytes are activated by an antigen signal, calcineurin dephosphorylates the nuclear factor of an activated T cell (NFAT), allowing it to translocate into the nucleus of the cell and regulate the transcription of certain genes involved in the immune response; however, in the presence of calcineurin inhibitors, NFAT remains phosphorylated and cannot translocate into the nucleus, inhibiting its ability to activate the genes necessary for immune system activation [156,157].

The inhibition of the calcineurin pathway can also have adverse effects on renal function. Furthermore, it has been well demonstrated in a series of mechanistic enzymatic experiments that ROS inhibits calcineurin [158].

Calcineurin inhibitors induce nephrotoxicity through reversible vasoconstriction in the afferent arteriole. Vasoconstriction results in subsequent relative hypoxia, progressive arteriolosclerosis, hyaline arteriolopathy, tubular atrophy, and interstitial fibrosis [159]. Indeed, calcineurin is implicated in the regulation of renal blood flow and the function of renal tubular cells, which are responsible for the filtration and reabsorption of substances in the kidneys. The inhibition of this pathway can cause a decrease in renal blood flow and glomerular filtration, leading to impaired renal function and CKD [160].

Studies suggest that certain pesticides could indirectly interact with cellular signaling pathways, including the calcineurin pathway. For example, it has been observed that all type II pyrethroid insecticides (such as cypermethrin and deltamethrin) are potent inhibitors of calcineurin [161]; however, the exact mechanisms of these interactions are not yet fully elucidated, and further research is needed for better understanding.

### 4.3. Point of View

Recent research findings provide compelling evidence supporting the hypothesis that CINAC is a kidney disease induced primarily by toxins. A recent study examined the effect of cyclosporine, a well-established immunosuppressive nephrotoxin, on the kidneys and found histopathological alterations similar to those observed in patients with CINAC [162]. In contrast, no significant correlation was found between dehydration and specific kidney damage observed in CINAC patients. These results reinforce the idea that exposure to toxins, rather than dehydration, is the main trigger for CINAC [162].

Furthermore, the rapid increase in CINAC incidence in the 1990s in Sri Lanka and Central America does not appear to be associated with abrupt variations in working conditions, temperatures, or precipitation [21,163]. Additionally, despite the existence of agricultural communities in warm environments, especially in the case of some villages near affected areas, no incidence of the disease was recorded. These findings suggest that other factors, in addition to heat, could also play a role in the origin of this epidemic.

The adverse effects of pesticides, including paraquat, glyphosate, and pyrethroids, on kidneys’ health have been well-approved, and their widespread use in crops coincides with the emergence of the CINAC epidemic in these regions [30].

The direct and perpetual exposition of farmers to various chemicals has many adverse effects on their organs’ health, especially their kidneys, which could be entirely damaged over time. Nevertheless, this disease can also affect people who are not directly exposed to agricultural working conditions, including women and children, suggesting non-occupational exposure to toxins through ingestion or inhalation, given that they share the same environment as their partners [164].

Overall, we believe that dehydration may be a contributing risk factor and may play a role in the development of kidney disease, but alone is not sufficient to be a direct cause of the CINAC; thus, other factors, such as exposure to toxins, might be more important.

## 5. Early Detection of CKDu

In patients with CKDu, protein excretion and urinary albumin/creatinine ratio (ACR), elevated serum creatinine, which appears only after significant renal tissue damage, are the main clinical manifestations; consequently, one of the major challenges in CKDu screening is the shortage in early diagnostic tests. Although testing kidney tissue samples is ideal because it is the affected tissue; however, obtaining biopsies is invasive, more expensive, and not suitable for initial screening purposes [165]. According to Ju et al. [166], a biological marker (biomarker) is a characteristic objectively measured and evaluated as an indicator of normal biological processes, pathogenic processes, or pharmacological responses to a therapeutic intervention [166]. In a clinical setting, biomarkers have a great potential to be used as tools for early detection, more effective treatment, and a more personalized approach to medical care [167]. They could be identified at any level of the genome–phenome continuum and could be genomic biomarkers DNA or bio-markers (proteins) or metabolic biomarkers (metabolites) [166].

Proteomic biomarkers represent a promising method to improve the management of patients with kidney disease by allowing more accurate and earlier detection of kidney pathology [168]. Especially in bio-fluids, mainly in the urine, serum, and plasma, which are much less invasive for patients. In addition, urine has a higher priority compared with other bio-fluid samples because it is a source of more specific biomarkers as it is in direct contact with the glomeruli, allowing the detection of changes in different peptide levels in patients with CKD [169]. Under normal physiological conditions, urine should contain only small amounts of low/medium molecular weight protein; this protein content evolves in pathological situations [170].

Peptides that represent short amino acids’ chains could also be in urines; however, their existence is issued from complex post-translational modifications of larger protein molecules [171]. Using capillary electrophoresis–MS, researchers compared urine samples from 230 patients with various kidney diseases and 379 controls with normal kidney function. In their study, researchers used a support vector machine to join 273 peptides into a classifier called CKD273 [172]. The main components of the CKD273 classifier in CKD patients include fragments of different collagens, kidney-specific proteins, and blood proteins, among other secreted proteins [173]. Some studies of peptidome in kidney disease reported an early reduction in the urinary abundance of collagen fragments I, III, and IV, indicating inhibition of the degradation of kidney collagen, which leads to a rise in the extracellular matrix and thus results in interstitial fibrosis [174].

**Figure 4:**
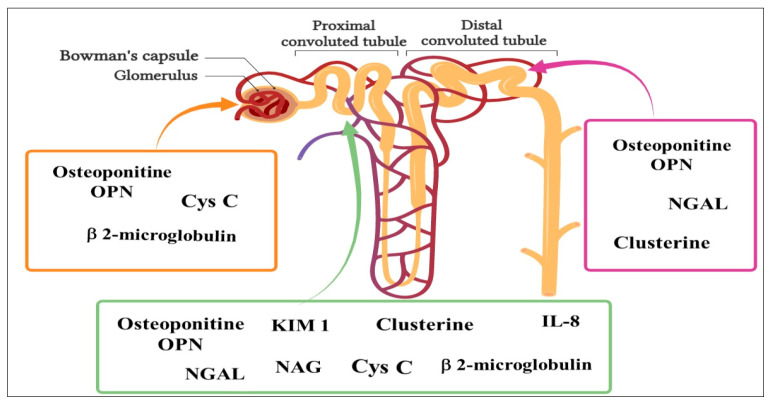
Kidney injury biomarkers.

In response to kidney injury, kidney tissues secrete several molecules at different locations (Figure 4), which could be used as biosensors to detect kidney disease in advance and help locate the injury site. These molecules include Kidney injury molecule-1 (KIM-1), α1-microglobulin (A1M), neutrophil gelatinase-associated lipocalin (NGAL), *N*-acetyl-beta-d-glucosaminidase (NAG), β2-macroglobulin (B2M), clusterin (CLU), IL-18 MCP-1, Cys *C*–cystatin, and osteopontin (OPN) [175].

KIM-1 is a type I transmembrane glycoprotein that is the most upregulated protein in the proximal tubule with any form of kidney injury that affects this nephron segment. It is recognized as a potential biomarker for the detection of tubular damage in major kidney diseases [176].

NGAL is a small circulating protein first found in neutrophil granules, which is highly modulated in a wide variety of disease situations, making it a useful biomarker of various disease states. Its use as a biomarker for AKI detection and progression has been thoroughly studied, as it is rapidly released after tubular injury [177].

A1M is a small multifunctional immunomodulatory protein that exists in the blood in its free form (or unrelated) [178]. Normal urine contains very small amounts of A1M, but in conditions with disturbed tubular function, reabsorption of A1M is reduced, and increased amounts are found in urine [179]; therefore, A1M is considered a marker of renal tubular dysfunction.

Β2M is a major histocompatibility complex class I molecule that is a biomarker of renal filtration and increased cell turnover; it is found on the surface of all nucleated cells and is particularly abundant in lymphocytes and monocytes [180]. Under normal physiological conditions, some Β2M may be secreted into the circulation from cell surfaces or as a result of the intracellular release and is cleared from the blood primarily by the kidneys [181].

NAG is a high molecular weight lysosomal enzyme that acts on glycosylated compounds. NAG is excreted in abnormally high amounts when renal tubular dysfunction leads to renal tubular epithelial cell damage. Urinary NAG can be used as an early urinary biomarker for the early detection and progression of nephropathy [182,183].

CLU is a multifunctional glycoprotein that has generated great controversy regarding its cellular roles in recent years. It plays important roles in protein homeostasis/proteostasis, the inhibition of cell death pathways, and the modulation of signaling and transcription networks [184,185].

OPN is a highly phosphorylated glycophosphoprotein with acidic characteristics and rich in aspartic acid. OPN, a multifunctional protein, has important functions, particularly in cardiovascular diseases, kidney stones, and in the process of inflammation, biomineralization, and cell viability [186]. OPN is mainly present in the loop of Henle and in the distal nephrons of normal kidneys in animals and humans. After kidney injury, OPN expression can be significantly upregulated in all tubular segments and glomeruli [187].

IL-8 is a pro-inflammatory cytokine that has been proposed as a marker for AKI. Interleukins are important mediators of the immune reaction in the response of innate immune system and adaptive immunity. It is filtered in inflammatory pathways by affecting monocyte/macrophage migration, monocyte and T-cell numbers, and osmosis [188]. Increased urinary MCP-1 levels correlate with the extent of interstitial inflammatory infiltrate [189].

The first study identified suspected cases of CKDu and detected early kidney damage in Sri Lankan farming communities using urinary biomarkers, showing that the prediction of tubular damage by urinary NGAL and KIM-1 was significantly correlated with high levels of urinary ACR [190].

An evaluation of renal biomarkers performance in urine of adult patients with CKDu, aimed to develop an ideal screening procedure. This study highlighted A1M as particularly effective in specific CKD detection. Moreover, findings indicated improved predictive accuracy with a combined biomarker panel (A1M + KIM1 + RBP4) for identifying disease groups [191].

In rural Salvadoran communities, a cross-sectional study was conducted to determine the prevalence of urinary markers of kidney damage, revealing particularly high levels of KIM-1, NAG, NGAL, and IL-18 in urine samples of the subjects having high adult mortality levels of CKDu. These results highlight the opportunity to use these molecules as early diagnostic tools [192]; however, none of the clinical studies published in India or Egyptian communities on CKDu appear to have used any of the new biomarkers.

The determination was based on conventional markers as a screening tool, and this can be explained by their prohibitive cost. In comparison, dialysis or kidney replacement remains an even greater challenge in developing countries. Early intervention may then be an effective strategy to minimize CKDu in affected populations and halt kidney damage [193]. In addition, the realization of genomic and proteomic analyses can help to understand the mechanisms of progression of a disease, including CKDu.

Genetic discoveries are increasingly being used to inform the clinical management of kidney disease and have improved diagnostics and disease surveillance [194]. Indeed, genetic diagnosis is important to understand the pathological mechanism by specifying the etiology of kidney disease [195]. The use of targeted exome sequencing (TES) to perform genetic diagnosis of 60 genes from patients with congenital anomalies of the kidneys and urinary tract (CAKUT) identified pathogenic nucleotide variants of five known pathogenic genes: HNF1B, PAX2, EYA1, UPK3A, and FRAS1 [196].

Moreover, the use of Next Generation Sequencing (NGS) techniques has significantly improved diagnostic yield and can be used to detect all types of genetic variants, from single nucleotide variants to large structural variants, for the early diagnosis of CKDu state [197]. A set of 73 genes has been proposed by the American College of Medical Genetics and Genomics (ACMG), many of which are associated with nephrology-relevant phenotypes (PALB2, GLA, HNF1A, MEN1, MAX, RET, SDHAF2, SDHB, SDHC, SDH, VHL, TMEM127, TSC1, TSC2, and WT1) [198].

Non-coding RNAs (ncRNAs), especially long non-coding RNAs (lncRNAs) such as HOTAIR, play a crucial role in gene regulation and influence the pathophysiology of kidney diseases [199,200]. Despite their clinical potential, the complexity of ncRNAs’ mechanisms of action requires further research for their exploitation as effective therapeutic and diagnostic tools. The investigation of early biomarkers to detect renal lesions seems to be a key point for the management of the main clinical renal diseases [201].

## 6. Treatment

To date, no fully specific and validated treatment exists for CKDu, complicating the management of this condition compared with other forms of chronic kidney disease. This is partly due to the unknown etiology of CKDu, which hinders the development of targeted therapies; however, several therapeutic approaches are particularly well-suited to slow the CKDu progression and effectively manage its symptoms.

Patients with advanced CKDu should plan for kidney transplantation or dialysis. Overall, transplantation remains the best modality offered to patients with ESKD since most of them are young or middle-aged [202]. If the donor lives in the same area as the patient, he may be at risk of developing CKDu, which requires follow-up after kidney donation [203].

Most patients with ESKD are unsuitable for kidney transplantation or have to wait a number of years on dialysis until kidney transplantation; therefore, hemodialysis (HD) remains the most common modality [204]; however, since rural communities represent the majority of the affected population, and the subjects live in areas far from HD units, some countries have adopted the “Peritoneal Dialysis” policy. Thus, patients can receive treatment at home without having to travel to hemodialysis centers two or three times a week [205].

So far, there are no guidelines for the management of patients with CKDu because of the lack of convincing evidence; however, some of the common practices of nephrologists include the use of allopurinol, potassium supplements, bicarbonate, corticosteroids, and sometimes the prescription of angiotensin receptor blockers and enzyme inhibitors. angiotensin conversion [206].

Allopurinol conferred substantial protection and enhanced kidney function in mice subjected to heat stress. Renal protection was linked to a decrease in intrarenal uric acid concentration and expression of heat shock proteins [207]; however, at high doses, allopurinol can induce kidney damage in rats [208]. Based on these data, to determine the reduction in uric acid in patients with CKDu, a clinical trial is highly recommended. In addition, sodium bicarbonate delays the progression of CKD [209], and its protective role in preventing kidney damage due to recurrent heat stress has been reported in several studies [209,210].

Metformin may also have beneficial effects on kidney function, kidney fibrosis, and inflammation in patients with type 2 diabetes [211]; however, the authors also point out that further research is necessary to establish the effectiveness of metformin in the treatment of chronic kidney disease and that its use should be supervised by a physician [212]. They note that some patients with chronic kidney disease may not be able to tolerate metformin due to gastrointestinal tolerance issues [211].

## 7. Prevention

Preventive measures focus primarily on risk factors, screening, and surveillance of at-risk populations. Early diagnosis and appropriate interventions are essential. Since there is no definite etiology, the presumed causative factors are potentially preventable. These prevention methods include:

### 7.1. Changing Agricultural Practices

The WHO and the Food and Agriculture Organization of the United Nations have issued strong recommendations, which include the improvement of environmentally friendly practices, mandatory provision of personal protective equipment (PPE), encouragement of organic farming as well as the control of the use and sale of agrochemicals.

### 7.2. Fight Against the Consumption of Alcohol and Tobacco

By reducing the use of nonsteroidal anti-inflammatory drugs (and other drugs such as paracetamol) as well as illegal alcohol and tobacco.

### 7.3. Environmental Protection

Air, soil, and water pollution resulting from agrochemicals and heavy metals contribute to increased levels of acid rain and the prevalence of certain chronic diseases.

The protection of natural resources and the investment in environmental protection would have an advantageous impact on socioeconomic circumstances and a decrease in chronic diseases, including CKDu.

### 7.4. Early Detection of the Disease

Develop a screening method based on tubular damage markers to detect early loss of kidney function, enhancing our ability to characterize high-risk populations and affected regions more effectively.

### 7.5. Research Program

Completion of GIS-based mapping (Geographic Information System) of the proposed causes of CKDu in affected regions as well as the use of a multidisciplinary method to combat this complicated disease, combining research and clinical collaboration in at-risk populations.

### 7.6. Effective Prospective Monitoring to Ensure Toxin-Free Drinking Water

The quality of drinking water was identified as the main development vector for CKDu; however, to reduce the incidence of this disease, water purification systems are one of the most cost-effective hand-held methods that produce clean water and remove all nephrotoxins, including agrochemicals, fertilizers, heavy metals, hardness, etc.

## 8. Conclusions

Chronic kidney disease is a non-communicable occupational disease responsible for worldwide morbidity and mortality, characterized by progressive loss of kidney function despite the absence of known risk factors such as diabetes, hypertension, glomerulonephritis, etc. This disease is characterized by a progressive and irreversible deterioration of kidney function, accompanied by nonspecific symptoms such as fatigue, weight loss, and sleep disturbances, which complicate diagnosis. In the absence of early intervention, CKDu progresses to advanced stages and requires costly treatments such as dialysis or kidney transplantation.

Some researchers have proposed that heat stress, caused by high temperatures and prolonged exposure to heat, could contribute to the development of CKDu. It is believed that dehydration due to excessive heat can lead to AKI and, over time, progress to chronic kidney disease; however, there is still a lack of scientific consensus on this relationship between them, and further researches are needed to determine the exact role of heat stress in CKDu. Additionally, exposure to agricultural chemicals, particularly pesticides, has also been suggested as a possible risk factor for CKDu. It is postulated that chronic exposure to these chemical substances could cause damage to the kidneys and contribute to the deterioration of renal function.

The common mechanism proposed between heat stress and toxins-induced renal injury is the ROS generation and oxidative stress. In heat stress, hyperosmolarity, aldose reductase activation, and polyol pathway activation lead to the production of ROS, which activates the inflammatory pathway, causing inflammation and damage to kidney tissues. Similarly, toxins absorbed through cellular transporters induce ROS increase in renal tissues through enzymatic complexes of the mitochondrial transport chain or electron flow. In both scenarios, the outcome of reactive oxygen species (ROS) generation and oxidative stress include inflammation, apoptosis, and necrosis of renal cells. These processes likely play a role in the initiation and development of CKDu. It is important to note that CKDu is a complex and multifactorial disease, and it likely results from the interaction of multiple factors.

The epidemiology of CKDu identifies the most affected areas and populations, thereby guiding the investigation of underlying molecular mechanisms. The high prevalence of the disease in certain specific regions suggests an association with environmental factors. Examining these mechanisms provides insight into how these elements contribute to disease progression. This understanding aids in developing treatment and prevention strategies tailored to local risks, focusing on exposure management and reduction in cellular damage.

Current research is limited by a lack of precise data on cumulative exposure to heat and pesticides, as well as the geographic variations of CKDu, complicating the generalization of findings. Rigorous epidemiological studies are necessary to determine the true prevalence and geographic distribution of CKDu, especially in regions still under-researched, such as Africa. Special attention should be given to potential interactions between environmental stressors and genetic susceptibilities.

Future perspectives include collaborative studies aimed at identifying early detection biomarkers, interventional research to test hypotheses on the causes of CKDu in at-risk populations, and proactive prevention strategies. Given current knowledge, it is imperative to implement awareness and prevention programs to mitigate the impact of this silent disease within vulnerable communities.
